# Efficient acquisition of iron confers greater tolerance to saline-alkaline stress in rice (*Oryza sativa* L.)

**DOI:** 10.1093/jxb/erw407

**Published:** 2016-11-03

**Authors:** Qian Li, An Yang, Wen-Hao Zhang

**Affiliations:** ^1^State Key Laboratory of Vegetation and Environmental Change, Institute of Botany, the Chinese Academy of Sciences, Beijing 100093, China; ^2^University of Chinese Academy of Sciences, Beijing 100049, China; ^3^Research Network of Global Change Biology, Beijing Institutes of Life Science, Chinese Academy of Sciences, Beijing 100093, China

**Keywords:** Dongdao-4, iron acquisition, iron deficiency, Jigeng-88, *Oryza sativa*, rice, saline-alkaline stress.

## Abstract

To elucidate the mechanisms underlying tolerance to saline-alkaline stress in two rice genotypes, Dongdao-4 and Jigeng-88, we exposed them to medium supplemented with 10 mM Na_2_CO_3_ and 40 mM NaCl (pH 8.5). Dongdao-4 plants displayed higher biomass, chlorophyll content, and photosynthetic rates, and a larger root system than Jigeng-88 under saline-alkaline conditions. Dongdao-4 had a higher shoot Na^+^/K^+^ ratio than Jigeng-88 under both control and saline-alkaline conditions. Dongdao-4 exhibited stronger rhizospheric acidification than Jigeng-88 under saline-alkaline conditions, resulting from greater up-regulation of H^+^-ATPases at the transcriptional level. Moreover, Fe concentrations in shoots and roots of Dongdao-4 were higher than those in Jigeng-88, and a higher rate of phytosiderophore exudation was detected in Dongdao-4 *versus* Jigeng-88 under saline-alkaline conditions. The Fe-deficiency-responsive genes *OsIRO2*, *OsIRT1*, *OsNAS1*, *OsNAS2, OsYSL2*, and *OsYSL15* were more strongly up-regulated in Dongdao-4 than Jigeng-88 plants in saline-alkaline medium, implying greater tolerance of Dongdao-4 plants to Fe deficiency. To test this hypothesis, we compared the effects of Fe deficiency on the two genotypes, and found that Dongdao-4 was more tolerant to Fe deficiency. Exposure to Fe-deficient medium led to greater rhizospheric acidification and phytosiderophore exudation in Dongdao-4 than Jigeng-88 plants. Expression levels of *OsIRO2*, *OsIRT1*, *OsNAS1*, *OsNAS2, OsYSL2*, and *OsYSL15* were higher in Dongdao-4 than Jigeng-88 plants under Fe-deficient conditions. These results demonstrate that a highly efficient Fe acquisition system together with a large root system may underpin the greater tolerance of Dongdao-4 plants to saline-alkaline stress.

## Highlight

A highly efficient iron acquisition system, together with a large root system, confers the greater tolerance of Dongdao-4 rice plants, relative to Jigeng-88 plants, to saline-alkaline stress.

## Introduction

Soil saline-alkalization is a major abiotic stress to agriculture worldwide, causing considerable damage to crop growth and loss of crop productivity (Martínez-Cuenca *et al.*, 2013). Based on Food and Agriculture Organization/UNESCO data, 831 million ha of soils are affected by saline-alkaline stress worldwide (Jin *et al.*, 2006). In northeast China, saline- alkaline soil, in which only a few species of alkali-resistant halophytic plants can survive, covers more than 6.2% (7.66 million ha) of the land area (Wang *et al.*, 2004). Moreover, soil alkalization in this region is continuing to expand, posing a serious threat to food production (Wang *et al.*, 2015*b*). Extensive studies have been conducted to elucidate the mechanisms by which plants respond and adapt to salinity resulting from increases in neutral salts in soils (Wang *et al.*, 2011; Zhang *et al.*, 2013). Accordingly, the molecular and physiological mechanisms underlying the response and adaptation to neutral salt stress are being dissected, particularly in the model plant species *Arabidopsis thaliana* and rice (Wang *et al.*, 2004, 2010, 2011; Zhang *et al.*, 2013). In contrast, fewer studies have been conducted to understand how plants respond and adapt to saline-alkaline stress due to enhanced accumulation of alkaline salts (i.e., Na_2_CO_3_ and NaHCO_3_) in soils. Plants suffering from salt stress resulting from high concentrations of neutral salts encounter problems associated with ionic toxicity and osmotic stress (Lv *et al.*, 2013). Saline-alkaline stress is more complex than neutral-salts stress: in addition to the presence of high levels of toxic Na^+^ and osmotic stress, high soil pH can also alter the availability of many mineral nutrients in soil, thus imposing nutrient stress upon plants (Shi and Wang, 2005). There are reports that plants respond differently to alkaline salts (Na_2_CO_3_ and NaHCO_3_) and neutral salts (NaCl and Na_2_SO_4_) (Yang *et al.*, 2007). For example, Guo *et al.* (2010) found that fructan synthesis in wheat seedlings was enhanced by saline treatment, while the fructan content was reduced when exposed to alkaline stress. Moreover, it has been shown that rice plants accumulated inorganic anions to maintain intracellular ionic equilibrium under salt stress, whereas the accumulation of organic acids (especially malate and citrate) was detected under alkaline stress (Wang *et al.*, 2015*b*). Elucidation of the mechanisms by which plants respond and adapt to saline-alkaline stress will therefore provide important clues for the selection and breeding of crop genotypes capable of growth in saline-alkaline soils.

Saline-alkaline soils are characterized by both high salinity and high alkalinity (soil pH often greater than 8.0) due to the hydrolysis of two sodium carbonates, NaHCO_3_ and Na_2_CO_3_ (Tang *et al.*, 2014). Plants have to face ionic toxicity, osmotic stress, and high pH stress simultaneously in saline-alkaline soils. Plants have evolved several mechanisms to adapt to the problems associated with saline-alkaline stress. These include extrusion and compartmentalization of toxic Na^+^ to maintain ion homeostasis (Wang *et al.*, 2012; Yang *et al.*, 2007), accumulation of osmoprotectants such as soluble sugars and glycine betaine to facilitate water uptake (Cha-Um *et al.*, 2009), and secretion of organic anions and H^+^ to acidify the rhizosphere (Liu *et al.*, 2010; Wang *et al.*, 2015*b*; Zamani Babgohari *et al.*, 2013). In addition, high soil pH reduces the solubility of iron (Fe) in the soil solution, and plants growing in alkaline soils often exhibit distinct symptoms of Fe deficiency chlorosis (Kakei *et al.*, 2012). In alkaline soils, Fe occurs mainly in the form of insoluble hydroxides and oxides, limiting its bioavailability for plants. Therefore, in addition to tolerance to saline stress, plants that can grow in saline-alkaline soils should have a greater capacity to acquire Fe, thus equipping them with tolerance to Fe deficiency in saline-alkaline growth medium.

Three general strategies are often used by higher plants for the acquisition of Fe: acidification, reduction, and chelation (Abadia *et al.*, 2011). The acidification of the rhizosphere resulting from H^+^-ATPase-mediated proton extrusion is important for Fe nutrition, as low rhizospheric pH can facilitate Fe solubilization (Santi and Schmidt, 2009). The reduction-based Strategy I mechanism operates in non-graminaceous plants, while the chelation-based Strategy II mechanism occurs in graminaceous plants (Ishimaru *et al.*, 2006; Kobayashi and Nishizawa, 2012; Schmidt, 2003). Strategy I plants are distinguished by their reduction of ferric iron (Fe^3+^) to ferrous iron (Fe^2+^) by ferric-chelate reductase, and uptake of Fe^2+^ into root cells by IRON REGULATED TRANSPORTER1 (IRT1) (Brumbarova *et al.*, 2015). Reduction of Fe^3+^ to Fe^2+^ is a rate-limiting step for Fe acquisition in Strategy I plants; this is an enzymatic process catalyzed by the FERRIC REDUCTASE-OXIDASE (FRO) family protein FRO2 (Robinson *et al.*, 1999). Three genes encoding Fe^3+^-chelate reductases, *FRO2*, *FRO1*, and *FRO3*, have been identified in Arabidopsis (Robinson *et al.*, 1997, 1999) and pea (Waters *et al.*, 2002). Two genes encoding Fe^2+^ transporters, *IRT1* (Bughio *et al.*, 2002) and *IRT2* (Ishimaru *et al.*, 2006), have been isolated in rice plants. The addition of bicarbonate to growth medium has been demonstrated to cause foliar chlorosis in *Parietaria diffusa*, a Strategy I plant, due to reduction in Fe acquisition (Donnini *et al.*, 2012). Lucena *et al.* (2007) reported that bicarbonate induced foliar chlorosis and inhibited the expression of genes encoding FER-like transcription factors, thus inhibiting the expression of ferric reductase, iron transporter, and H^+^-ATPase genes in a number of Strategy I plants. Strategy II plants can synthesize and exude phytosiderophores belonging to the mugineic acid (MA) family to chelate Fe^3+^ and then take up the Fe^3+^–MAs complex by specific transporters under Fe-deficient conditions (Araki *et al.*, 2015; Jolley and Brown, 1989). MAs are synthesized from S-adenosyl-L-methionine in a process catalyzed sequentially by three enzymes: nicotianamine synthase (NAS), nicotianamine aminotransferase (NAAT), and deoxymugineic acid synthase (DMAS) (Itai *et al.*, 2013; Wang *et al.*, 2015*a*; Yang *et al.*, 2013). Transporters responsible for Fe acquisition and allocation and transcription factors involved in the regulation of Fe homeostasis are being identified (Kobayashi and Nishizawa, 2012; Yang *et al.*, 2013). Despite classification of the mechanisms for Fe acquisition by plants into Strategy I and Strategy II, the two mechanisms cannot be mutually exclusive. For example, enhanced uptake of Fe^2+^ by IRT1 and Fe^3+^–MAs by rice plants has been observed under Fe-deficient conditions (Ishimaru *et al.*, 2007). Moreover, monocot *Puccinellia tenuiflora* and *Poa annua* plants differ in their tolerance to saline-alkaline stress (Kobayashi *et al.*, 2015). By RNA-sequencing analysis, Kobayashi *et al.* (2015) found that homologs of the genes involved in Fe acquisition were up-regulated in *P. tenuiflora* and down-regulated in *P. annua* when challenged by NaHCO_3_. These results suggest that efficient Fe acquisition by plants growing in saline-alkaline soils is an important trait to enable them to tolerate saline-alkaline stress.

Rice (*Oryza sativa* L.), a major staple food for the world’s population, is salt-sensitive and moderately tolerant to sodicity (Wang *et al.*, 2004). Rice is often used as a desalinization crop in alkaline soils (Lv *et al.*, 2013). However, in contrast to salt stress, few studies have evaluated the mechanisms underlying saline-alkaline stress in rice plants. Different species and genotypes within a species can differ in their tolerance to saline-alkaline stress (Ksouri *et al.*, 2005). In our previous studies, we collected more than 100 rice genotypes and assessed their tolerance to saline-alkaline stress. Among the rice genotypes screened, we found that the genotype Dongdao-4 was the most tolerant to saline-alkaline stress, while the genotype Jigeng-88 was the most sensitive. These two genotypes were therefore used in this study to dissect the physiological and molecular mechanisms responsible for saline-alkaline tolerance in rice plants.

## Materials and methods

### Plant materials, growth conditions, and stress treatments

Two rice genotypes, *O*. *sativa* L. ssp. *Japonica* cv. Dongdao-4 and Jigeng-88, were used in this study. Seeds were germinated in tap water at 37 °C for 2 days and transferred on to moist tissue paper for 2 days at 30 °C in the dark. The germinated seedlings were then transferred to nutrient solution containing 1.425 mM NH_4_NO_3_, 0.42 mM NaH_2_PO_4_, 0.510 mM K_2_SO_4_, 0.998 mM CaCl_2_, 1.643 mM MgSO_4_, 0.168 mM Na_2_SiO_3_, 0.125 mM Fe-EDTA, 0.019 mM H_3_BO_3_, 0.009 mM MnCl_2_, 0.155 mM CuSO_4_, 0.152 mM ZnSO_4_, and 0.075 mM Na_2_MoO_4_. This hydroponic experiment was carried out in a growth chamber maintained at 30 °C/22 °C (day/night) with a 14-h photoperiod and relative humidity maintained at approximately 70%.

For analysis of tolerance to saline-alkaline stress, Dongdao-4 and Jigeng-88 plants were grown in the culture solution for 3 weeks. Then, half of the plants were transferred to culture solution supplemented with 40 mM Na^+^ (10 mM Na_2_CO_3_ and 20 mM NaCl) for 1 day and then exposed to 60 mM Na^+^ (10 mM Na_2_CO_3_ and 40 mM NaCl) for 1 week. The remaining plants were kept in the original culture solution as a control. The solution was refreshed on a daily basis. The pH of the control hydroponic solution was adjusted to 6.0, while the solution used for saline-alkaline treatment was adjusted to pH 8.5.

To evaluate the effects of Fe deficiency on the two rice genotypes, Dongdao-4 and Jigeng-88 seedlings were cultured in the control hydroponic solution for 2 weeks and then transferred to a hydroponic solution supplemented with 0.01 μM Fe-EDTA for 2 weeks.

### Measurements of plant growth

Shoots and roots of the rice seedlings were harvested and oven-dried at 75 °C for 2 days until they reached constant mass. To analyze root morphological parameters, roots were scanned with an Epson digital scanner (Expression 10000XL, Epson Inc.) and scans were analyzed with WinRHIZO/WinFOLIA software (Regent Instruments Inc.).

### Determination of metal content

Shoots and roots of rice seedlings were harvested and dried as described above. They were ground into fine powder and digested with 6 mL nitric acid and 2 mL perhydrol using a microwave system (MARS, CEM). Total metal content was determined by inductively coupled plasma mass spectrometry (ICAP6300; Thermo Scientific, Waltham, MA, USA).

### Measurements of chlorophyll concentration

To determine chlorophyll concentration in Dongdao-4 and Jigeng-88 plants, newly formed leaves were harvested, weighed, and extracted with aqueous ethanol (95% v/v). The absorbance (A) of the supernatant was recorded at wavelengths of 663 and 645 nm. Total chlorophyll concentration was calculated as 8.02*A*_663_+20.21*A*_645_, and was expressed as mg chlorophyll g^–1^ fresh weight. Total chlorophyll concentration values (SPAD values) were also determined on the fully expanded youngest leaves of seedlings with a portable chlorophyll meter (SPAD-502, Minolta Sensing).

### Measurements of photosynthetic characteristics

Photosynthetic rates of rice seedlings were measured between 8.30 and 11.30 h with a LI-6400 XT portable photosynthesis system equipped with a LED leaf cuvette (Li-Cor, Lincoln, NE, USA). Artificial illumination was applied to the leaves in the chamber from a red–blue 6400-02B LED light source attached to the sensor head giving continuous light (1000 μmol m^–2^ s^–1^ photosynthetic photon flux density); ambient CO_2_ concentration was ~500 μmol CO_2_ mol^–1^. At least 15 individual Dongdao-4 and Jigeng-88 plants in each stress treatment were selected for measuring photosynthetic rates.

### Measurement of external solution pH

To measure root-induced pH changes, pH in the growth medium was monitored continuously starting at 9:00 h, using a pH-sensitive microelectrode (pH-HJ90B, Shanghai). In the saline-alkaline stress experiment, the solution was refreshed on a daily basis. The pH of the control hydroponic solution was adjusted to 5.9, while the solution used for saline-alkaline stress treatment was adjusted to pH 8.5. In the Fe-deficient stress experiment, the pH of the medium was adjusted to 5.9 and the solution was renewed every 4 days.

### Visualization and measurement of ferric reductase activity

Intact rice roots were thoroughly washed with saturated CaSO_4_ (pH 5.9) and then spread on an agar sheet containing 0.75% (w/v) agar, 0.1 mM Fe^3+^-EDTA, 0.01 M MES buffer (pH 5.9), and 0.3 mM ferrozine. The incubation for visualization of ferric reductase was conducted in a growth chamber under light. After 1 d incubation, ferric reductase-induced color changes along the roots become visible; a purple color indicated that Fe^3+^ was reduced to Fe^2+^ by ferric reductase and chelated by ferrozine.

For measuring ferric reductase activity, roots of Dongdao-4 and Jigeng-88 plants were immersed in saturated CaSO_4_ solution. After washing three times with deionized water, the roots were transferred to 20 mL of nutrient solution containing 0.1 mM Fe^3+^-EDTA, 10 mM MES buffer (pH 5.9), and 0.3 mM ferrozine for 1 h incubation in a growth chamber under light. The absorbance of the solution at 562 nm was measured. Ferric reductase activity was calculated based on the absorbance and expressed as μmol Fe^2+^ g^–1^ FW root h^–1^.

### Determination of root phytosiderophore release

Roots of Dongdao-4 and Jigeng-88 plants were rinsed thoroughly with deionized water after immersion in saturated CaSO_4_ solution, then placed in 40 mL of aerated deionized water for 2 h, to collect the root exudates at 12.00 h. Phytosiderophores were indirectly quantified by calculating the amount of Fe^3+^ mobilized from Fe hydroxide, following protocols described by Inal *et al.* (2007). In brief, an aliquot of 0.5 mL 1mM FeCl_3_ was added to 9 mL of the root exudates and the mixture was incubated for 1 h with shaking to produce Fe^3+^-phytosiderophore compounds. An aliquot of 1 mL 0.5 M sodium acetate (pH 7.0) was added to the mixture, which was shaken for another 15 min to precipitate the remaining Fe^3+^. The solution was then filtered. The filtrate was acidified by adding 0.2 mL 1.5 M H_2_SO_4_ together with 0.5 mL 1.15 M hydroxylammonium chloride and then incubated for 20 min at 55 °C, in order to convert Fe^3+^-phytosiderophore to Fe^2+^-phytosiderophore. An aliquot of 90 μL 0.03 M ferrozine was added to the filtrate to chelate the reduced Fe-phytosiderophore complex. The absorption of the filtrate at 562 nm was recorded and the phytosiderophore release rates were calculated as Fe equivalents.

### RNA isolation and real-time RT-PCR

Total RNA of leaves and roots was isolated using RNAiso reagent (Takara) and reverse transcribed into first-strand cDNA with a PrimeScript^®^ RT Reagent kit (Takara). Real-time PCR was performed in an optical 96-well plate with an Applied Biosystems Stepone TM Real-Time PCR system. Each reaction contained 7.5 μl of 2× SYBR Green Master Mix reagent, 0.5 μl of cDNA samples, and 0.6 μl of 10 μM gene-specific primers in a final volume of 15 μL. The thermal cycle used was as follows: 95 °C for 10 min, followed by 40 cycles of 95 °C for 30 s, 60 °C for 30 s, and 72 °C for 30 s. The following primers were used: *OsIRO2*, 5ʹ-CTCCCATCGTTTCGGCTACCT-3ʹ and 5ʹ-GCTGGGCACTCCTCGTTGATC-3ʹ; *OsIRT1*, 5ʹ-CGTC TTCTTCTTCTCCACCACGAC-3ʹ and 5ʹ-GCAGCTGATGATCG AGTCTGACC-3ʹ; *OsNAS1*, 5ʹ-GTCTAACAGCCGGACGATCGA AAGG-3ʹ and 5’-TTTCTCACTGTCATACACAGATGGC-3ʹ; *OsNAS2*, 5ʹ-TGAGTGCGTGCATAGTAATCCTGGC-3ʹ and 5ʹ-CA GACGGTGACAAACACCTCTTGC-3ʹ (Inoue *et al.* 2003); *OsYSL15*, 5ʹ-ACTGGTACCCTGCAAACATAC-3ʹ and 5ʹ-GCAAT GATGCTTAGCAAGAAG-3ʹ; Os12g0638700, 5ʹ-CTTGCCTCT GCTGTTTACCT-3ʹ and 5ʹ-GCTTCACAACCGATTCTACAT-3ʹ; Os03g0100800, 5ʹ-GCTGTTGCCTATCAGGAAGT-3ʹ and 5ʹ-TCA AAGAGTGGGAGAAGACC-3ʹ; Os03g0689300, 5ʹ-AAACGATG CTCCAGCCCTAA-3ʹ and 5ʹ-AATGGCGGGAAATCAAACT C-3ʹ; and *actin*, 5ʹ-ACCACAGGTATTGTGTTGGACTC-3ʹ and 5ʹ-AGAGCATATCCT TCATAGATGGG-3ʹ. Amplification of *actin* (GenBank accession no. AB047313) was used as an internal control. The relative expression level was analyzed by the comparative Ct method.

### Statistical analysis

All data were analyzed by ANOVA using SAS statistical software. Significant differences were evaluated using Student’s *t*-test.

## Results

### Effects of saline-alkaline stress on growth

**Fig. 1.**
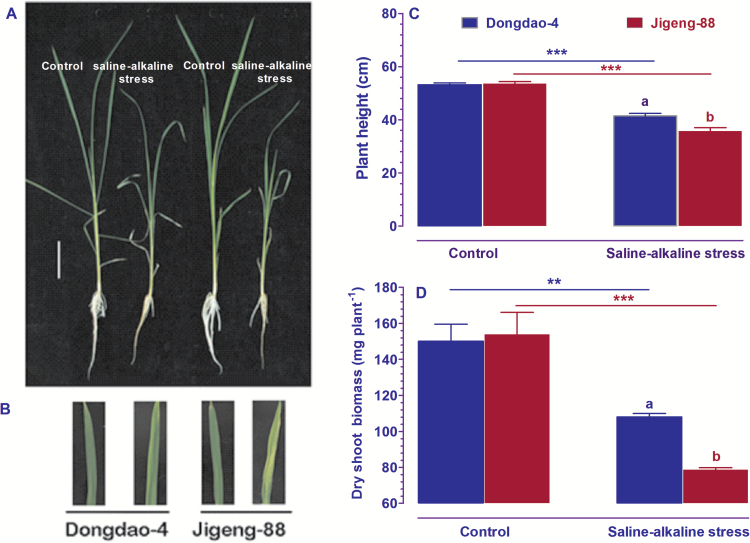
Effects of saline-alkaline stress on Dongdao-4 and Jigeng-88 seedlings. (A) Growth performance. (B) Photographs of the leaves corresponding to the seedlings in panel (A). (C) Plant height. (D) Dry shoot biomass. Three-week-old rice seedlings grown in normal culture solution were transferred to culture solution supplemented with 40 mM Na^+^ (10 mM Na_2_CO_3_ and 20 mM NaCl) for 1 day, and then exposed to 60 mM Na^+^ (10 mM Na_2_CO_3_ and 40 mM NaCl) for 1 week. Bars=10 cm. Data are means±SE (*n*≥4). Means with different letters are significantly different (*P*<0.05) within the same treatment. Asterisks indicate significant differences between control and saline-alkaline stress of the same genotype, as determined by Student’s *t*-test (*P<0.05, **P<0.01, ***P<0.001).

To compare the tolerance of Dongdao-4 and Jigeng-88 plants to saline-alkaline stress, 3-week-old seedlings of the two rice genotypes were exposed to growth solution supplemented with 60 mM Na^+^ and at pH 8.5 for 1 week. As shown in Fig. 1A, no differences in the growth of the two genotypes were detected when they were grown in the control solution. Upon exposure of the two genotypes to saline-alkaline medium, foliar chlorosis was observed in Jigeng-88, but not Dongdao-4 (Fig. 1B). In addition, the saline-alkaline stress treatment led to suppression of shoot and root growth in both genotypes; the magnitude of reduction in shoot and root growth of Dongdao-4 plants was significantly less than that in Jigeng-88 plants, such that plant height and dry shoot biomass of Dongdao-4 seedlings were significantly higher than those of Jigeng-88 seedlings (Fig. 1C, D).

**Fig. 2.**
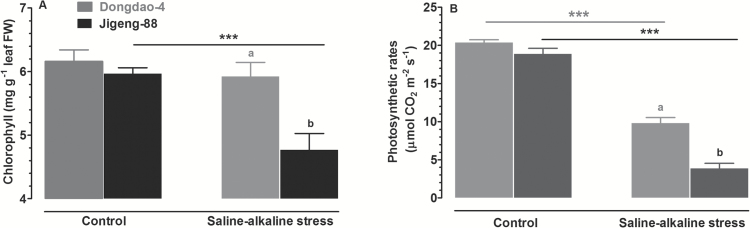
(A) Foliar chlorophyll concentration and (B) photosynthetic rates of Dongdao-4 and Jigeng-88 plants grown in normal and saline-alkaline stress conditions. Treatments and statistical analysis were as described in Fig. 1.

To explore the physiological mechanism by which Dongdao-4 plants exhibited greater growth than Jigeng-88 plants under saline-alkaline conditions, we compared the effects of saline-alkaline stress on foliar chlorophyll concentration and photosynthetic rates (Pn) of the two genotypes. Both chlorophyll concentration and Pn of the two genotypes were comparable under control conditions (Fig. 2A, B). Exposure to saline-alkaline medium led to a significant reduction in foliar chlorophyll concentration in Jigeng-88 seedlings, while Dongdao-4 plants maintained a relatively constant foliar chlorophyll concentration when challenged by saline-alkaline stress, leading to a significant higher chlorophyll concentration in Dongdao-4 plants (Fig. 2A). Similarly, there were no differences in Pn in the two genotypes under control conditions (Fig. 2B). There were significant reductions in Pn in both genotypes after exposure to saline-alkaline stress, but the magnitude of the reduction in Pn was greater in Jigeng-88 than in Dongdao-4, leading to a significantly higher Pn in Dongdao-4 (Fig. 2B). These results suggest that Dongdao-4 seedlings are more tolerant to saline-alkaline stress than Jigeng-88 seedlings.

### Effect of saline-alkaline stress on Na^+^ and K^+^ concentrations

**Fig. 3.**
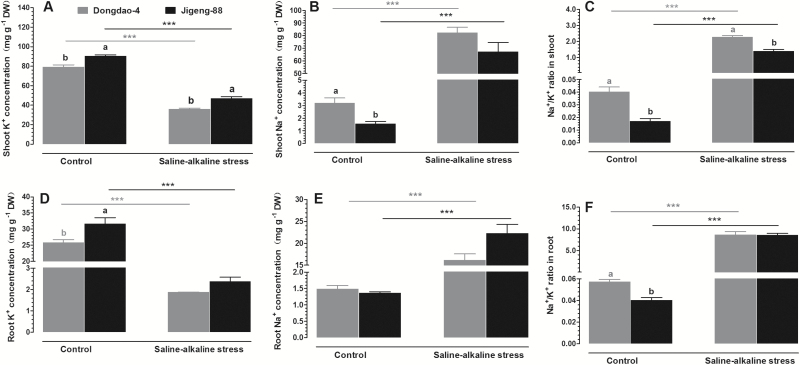
Effects of saline-alkaline stress on (A, D) K^+^ concentration, (B, E) Na^+^ concentration, and (C, F) Na^+^/K^+^ ratio of shoots and roots in Dongdao-4 and Jigeng-88 seedlings grown in normal and saline-alkaline stress conditions. Treatments and statistical analysis were as described in Fig. 1.

Given that rice plants suffering from saline-alkaline stress have to cope with excess toxic Na^+^ in the growth substrate, we compared the effects of saline-alkaline stress on Na^+^ and K^+^ concentrations as well as Na^+^/K^+^ ratios in shoots and roots of the two genotypes. Shoots of Dongdao-4 plants showed a lower K^+^ concentration and higher Na^+^ concentration than Jigeng-88 plants under control, non-saline-alkaline conditions (Fig. 3A, B). Exposure to saline-alkaline stress led to a significant increase in Na^+^ concentration and reduction in K^+^ concentration in shoots of both Dongdao-4 and Jigeng-88 plants (Fig. 3A, B). These changes resulted in a higher K^+^ concentration in shoots of Jigeng-88 than in shoots of Dongdao-4, whereas no significant differences in Na^+^ concentration in shoots between the two genotypes were found under saline-alkaline conditions (Fig. 3A, B). The greater increase in Na^+^ concentration and decrease in K^+^ concentration in shoots of Dongdao-4 led to a higher Na^+^/K^+^ ratio in Dongdao-4 than in Jigeng-88 plants (Fig. 3C). Under control conditions, a higher K^+^ concentration in roots of Jigeng-88 than in roots of Dongdao-4 was detected, while Na^+^ concentrations in roots of both genotypes were comparable (Fig. 3D, E). There were significant increases in root Na^+^ concentrations and reductions in root K^+^ concentrations of the two genotypes upon exposure to saline-alkaline medium, leading to comparable K^+^ and Na^+^ concentrations in the roots of the two genotypes (Fig. 3D, E). A higher Na^+^/K^+^ ratio in roots of Dongdao-4 than Jigeng-88 plants grown in control medium was found (Fig. 3F). The increases in root Na^+^ concentration and decreases in K^+^ concentration associated with saline-alkaline treatment led to significant increases of Na^+^/K^+^ ratio in the roots of both genotypes; the Na^+^/K^+^ ratio of the two genotypes did not differ significantly under saline-alkaline stress (Fig. 3F).

### Effect of saline-alkaline stress on Fe concentration

**Fig. 4.**
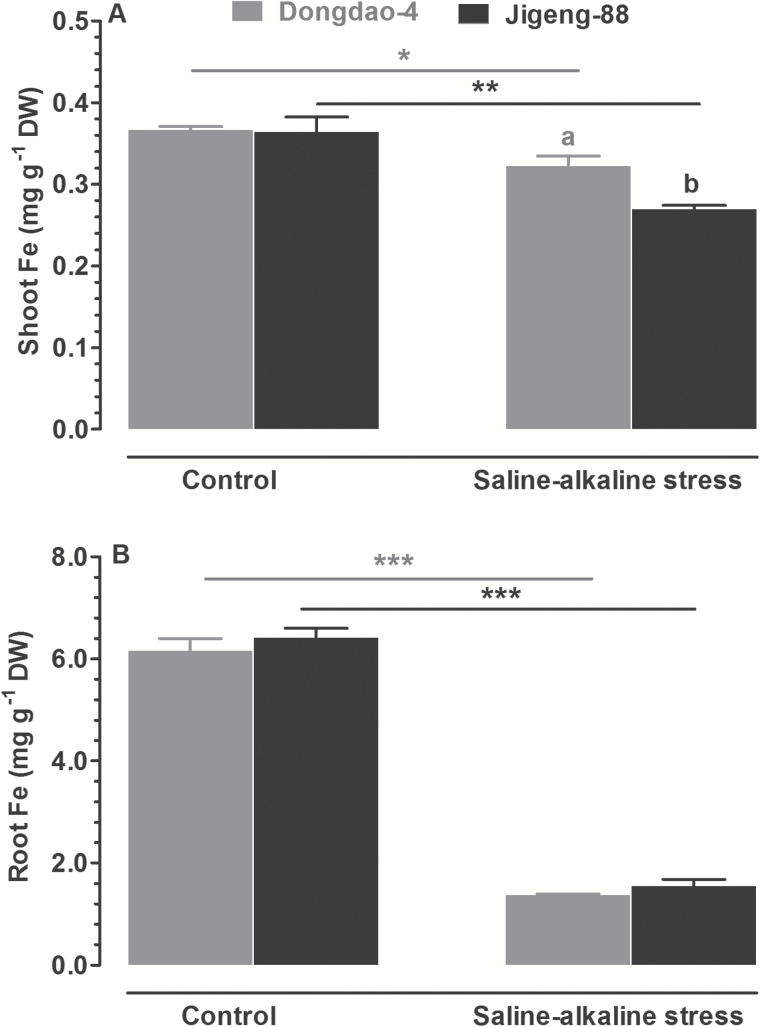
Fe concentrations of (B) shoots and (B) roots of Dongdao-4 and Jigeng-88 seedlings grown in normal and saline-alkaline stress conditions. Treatments and statistical analysis were as described in Fig. 1.

The concentration of plant-available Fe in saline-alkaline soils is often low due to immobilization, and this often becomes a limiting factor for crop yield and quality. Therefore, plants that are tolerant to saline-alkaline soils may also be equipped with an efficient Fe acquisition system. To test this hypothesis, we measured shoot Fe concentrations in the two genotypes grown in control and saline-alkaline media. The two genotypes exhibited comparable shoot Fe concentrations when grown in control medium (Fig. 4A). Upon exposure to saline-alkaline medium there was a significant reduction in shoot Fe concentration of Jigeng-88 seedlings, whereas shoot Fe concentration in Dongdao-4 seedlings remained relatively unchanged, leading to a significantly higher shoot Fe concentration in Dongdao-4 plants (Fig. 4A). The two genotypes showed comparable root Fe concentrations when grown in control medium. Exposure to saline-alkaline solution led to similar reductions in root Fe concentrations in the two genotypes, such that no significant difference was found between the genotypes (Fig. 4B).

### Effects of saline-alkaline stress on root system architecture

**Fig. 5.**
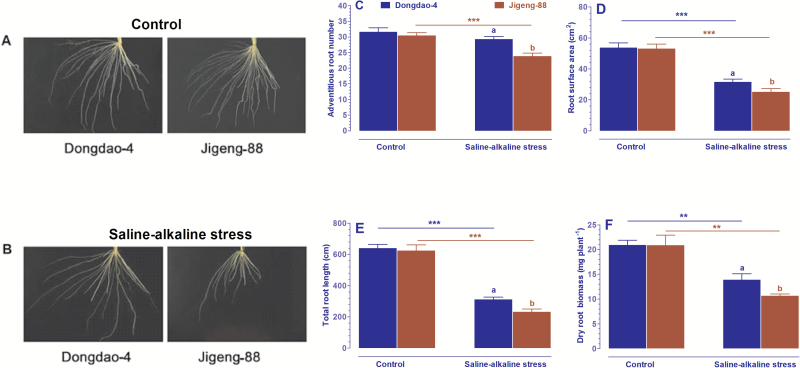
Effects of saline-alkaline stress on the root system architecture of Dongdao-4 and Jigeng-88 plants. (A) Root phenotypes of the two cultivars under normal conditions. (B) Root phenotypes of the two cultivars under saline-alkaline stress. (C) Adventitious root number. (D) Root surface area. (E) Total root length. (F) Dry root biomass. Treatments and statistical analysis were as described in Fig. 1.

Root system architecture is an important trait responsible for efficient Fe acquisition under Fe-deficient conditions. The observation that Dongdao-4 seedlings had a higher foliar Fe concentration than Jigeng-88 seedlings under saline-alkaline conditions prompted us to evaluate whether the root systems of the two genotypes differed in their response to saline-alkaline treatment. As shown in Fig. 5, exposure of the two genotypes to saline-alkaline medium led to significant reductions in their adventitious root number and root surface area; the reduction was greater in Jigeng-88 than in Dongdao-4 plants (Fig. 5C, D), such that Dongdao-4 seedlings exhibited significantly greater adventitious root number and root surface area. The root system of the two genotypes was comparable when grown in the control solution; the total root length and dry root biomass of Dongdao-4 and Jigeng-88 seedlings were comparable under control conditions. Both parameters were markedly reduced in the two genotypes by saline-alkaline treatment, with a greater reduction in Jigeng-88 than in Dongdao-4 plants, leading to a significantly longer total root length and higher dry root biomass in Dongdao-4 plants (Fig. 5E, F). These results suggest that the larger root system of Dongdao-4 seedlings under saline-alkaline stress may facilitate their Fe acquisition, thus providing them with tolerance to Fe deficiency.

### Effect of saline-alkaline stress on Fe deficiency-induced acidification of the rhizosphere

**Fig. 6.**
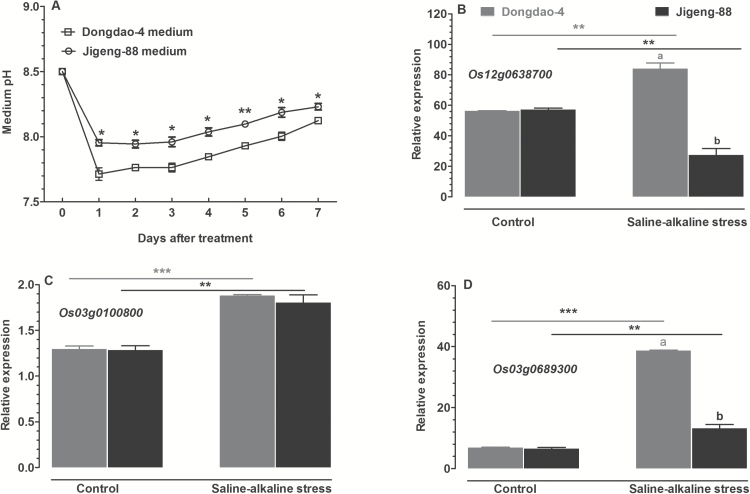
Effects of saline-alkaline stress on root acidification. (A) Time course of changes in the pH of the growth solution when intact rice roots were incubated for 7 days under control and saline-alkaline stress conditions. The solution was refreshed on a daily basis and its pH was adjusted to 8.5. (B–D) Expression patterns of the plasma membrane H^+^-ATPase genes (B) *Os12g0638700*, (C) *Os03g0100800*, and (D) *Os03g0689300* in roots of Dongdao-4 and Jigeng-88 plants grown in control and saline-alkaline medium for 7 days. Data are means±SE (*n*≥3). Means with different letters are significantly different (*P*<0.05) within the same treatments. Asterisks indicate significant differences between control and saline-alkaline stress treatments of the same genotype, as determined by Student’s *t*-test (*P<0.05, **P<0.01, ***P<0.001).

Acidification of the rhizosphere can facilitate Fe acquisition by plants. To test whether the two rice genotypes differed in their capacity to acidify the rhizosphere, we compared the effects of saline-alkaline stress on rhizosphere acidification by monitoring changes in medium pH. On exposure to saline-alkaline stress, a significantly greater reduction in growth medium pH was observed for Dongdao-4 plants relative to Jigeng-88 plants (Fig. 6A).

We further investigated the effect of saline-alkaline stress on transcripts of H^+^-ATPases in the two genotypes. No differences in levels of the three transcripts encoding rice H^+^-ATPases (*Os12g0638700*, *Os03g0100800*, and *Os03g0689300*) were detected between Dongdao-4 and Jigang-88 plants grown in control medium (Fig. 6B–D). The expression levels of the three genes were markedly up-regulated by saline-alkaline treatment in Dongdao-4 (Fig. 6B–D). In contrast, expression of *Os12g0638700* and *Os03g0689300* in Jigeng-88 was suppressed by the saline-alkaline treatment, leading to significantly higher expression levels for these two genes in Dongdao-4 plants (Fig. 6B–D).

### Effect of saline-alkaline stress on secretion of phytosiderophores

**Fig. 7.**
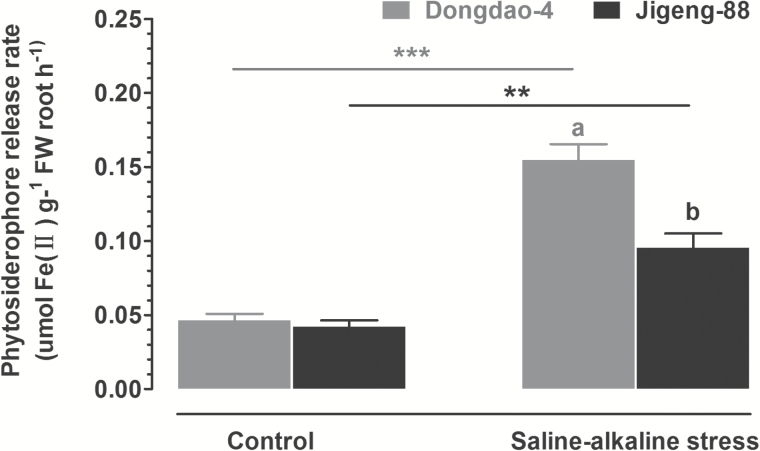
Effects of saline-alkaline stress on phytosiderophore release from roots of Dongdao-4 and Jigeng-88. Data (means±SE; *n*≥3) were collected after exposure of the rice plants to saline-alkaline medium for 7 days. Means with different letters are significantly different (*P*<0.05) within the same treatment. Asterisks indicate significant differences between control and saline-alkaline stress treatments of the same genotype, as determined by Student’s *t*-test (**P<0.01, ***P<0.001).

To test whether the two genotypes differ in their capacity to exude phytosiderophores under Fe-deficient conditions, we compared the effects of saline-alkaline stress on their phytosiderophore release rates. The two genotypes had comparable low levels of phytosiderophore release rates in control medium. Exposure to the saline-alkaline medium led to significant increases in phytosiderophore release in both genotypes; the increase in Dongdao-4 was greater than in Jigeng-88 (Fig. 7).

### Effects of saline-alkaline stress on expression of Fe deficiency-related genes

**Fig. 8.**
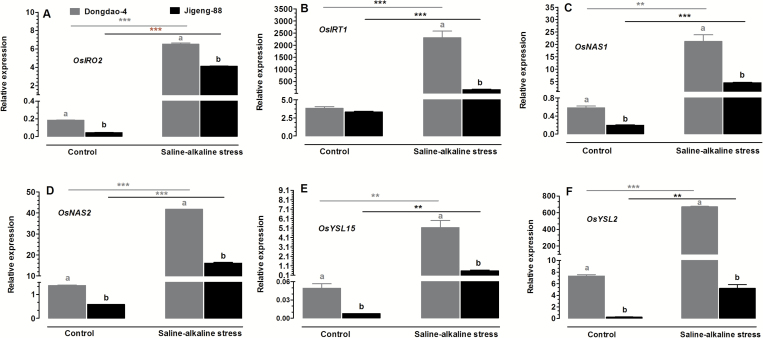
Effects of saline-alkaline stress on the expression of six Fe-deficiency-related genes in Dongdao-4 and Jigeng-88 plants: (A) *OsIRO2*, (B) *OsIRT1*, (C) *OsNAS1*, (D) *OsNAS2*, (E) *OsYSL15*, and (F) *OsYSL2*. Total RNA was extracted from rice seedlings grown under control and saline-alkaline stress conditions for 1 week. Transcript levels were measured by real-time RT-PCR. Actin was used as an internal control. The transcript levels of *OsIRT1* and *OsYSL2* were multiplied by 1000. Error bars are calculated based on three biological replicates. Treatments and statistical analysis were as described in Fig. 1.

The greater capacity of the Dongdao-4 genotype to acquire Fe under saline-alkaline conditions suggests that it is more tolerant to Fe deficiency than Jigeng-88. To further explore the physiological mechanisms underlying the greater accumulation of Fe in Dongdao-4 plants, we monitored changes in the expression patterns of genes involved in Fe acquisition, including *OsIRO2*, *OsIRT1*, *OsNAS1*, *OsNAS2*, *OsYSL15*, and *OsYSL2*. Among these genes, the expression levels in Dongdao-4 were greater than in Jigeng-88 under both control and saline-alkaline conditions, except for *OsIRT1*, which exhibited a comparable expression level in the two genotypes in control medium, and a higher expression level in Dongdao-4 than in Jigeng-88 in saline-alkaline medium (Fig. 8). Expression levels of the six genes were markedly up-regulated by saline-alkaline treatment; the treatment-induced up-regulation was greater in Dongdao-4 than in Jigeng-88, such that a significantly higher expression level in Dongdao-4 plants was observed under saline-alkaline conditions (Fig. 8).

### Effect of Fe deficiency on Dongdao-4 and Jigeng-88

**Fig. 9.**
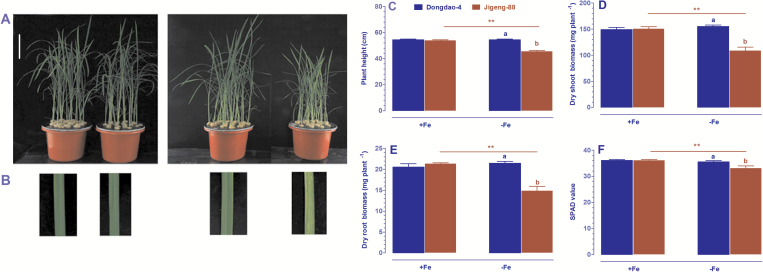
Physiological responses of the Dongdao-4 and Jigeng-88 rice genotypes to different Fe levels. (A) Growth performance. (B) Photographs of leaves corresponding to the seedlings in panel (A). (C) Plant height. (D) Dry shoot biomass. (E) Dry root biomass. (F) Total chlorophyll (SPAD) values. Two-week-old rice seedlings were exposed to Fe-sufficient (+Fe) or Fe-deficient (–Fe) medium for 2 weeks. Bars=10 cm. Data are means±SE (*n*≥3). Means with different letters are significantly different (*P*<0.05) within the same treatments. Asterisks indicate significant differences between +Fe and –Fe treatment of the same genotype, as determined by Student’s *t*-test (*P<0.05, **P<0.01, ** *P*<0.001).

The observation that Dongdao-4 plants can maintain Fe homeostasis under saline-alkaline conditions suggests that the Dongdao-4 genotype is highly efficient at Fe acquisition. To test this hypothesis, the effects of Fe deficiency on Dongdao-4 and Jigeng-88 plants were evaluated and compared. No differences in phenotype were observed when the two genotypes were grown in Fe-sufficient medium. Growth of Jigeng-88 was significantly suppressed upon transfer into Fe-deficient medium; in contrast, the growth of Dongdao-4 was unchanged by exposure to Fe-deficient medium (Fig. 9A). Jigeng-88, but not Dongdao-4, exhibited foliar chlorosis when exposed to Fe deficiency (Fig. 9B). In addition, there were no differences in the height, shoot biomass, root biomass, or SPAD value of Dongdao-4 plants grown in Fe-sufficient *versus* Fe-deficient solution. In contrast, Jigeng-88 plants exhibited significant reductions in height, shoot biomass, root biomass, and SPAD value when exposed to Fe-deficient medium (Fig. 9C–F). These results confirm that Dongdao-4 plants are more tolerant to Fe deficiency than Jigeng-88 plants.

### Effects of Fe deficiency on Fe accumulation

**Fig. 10.**
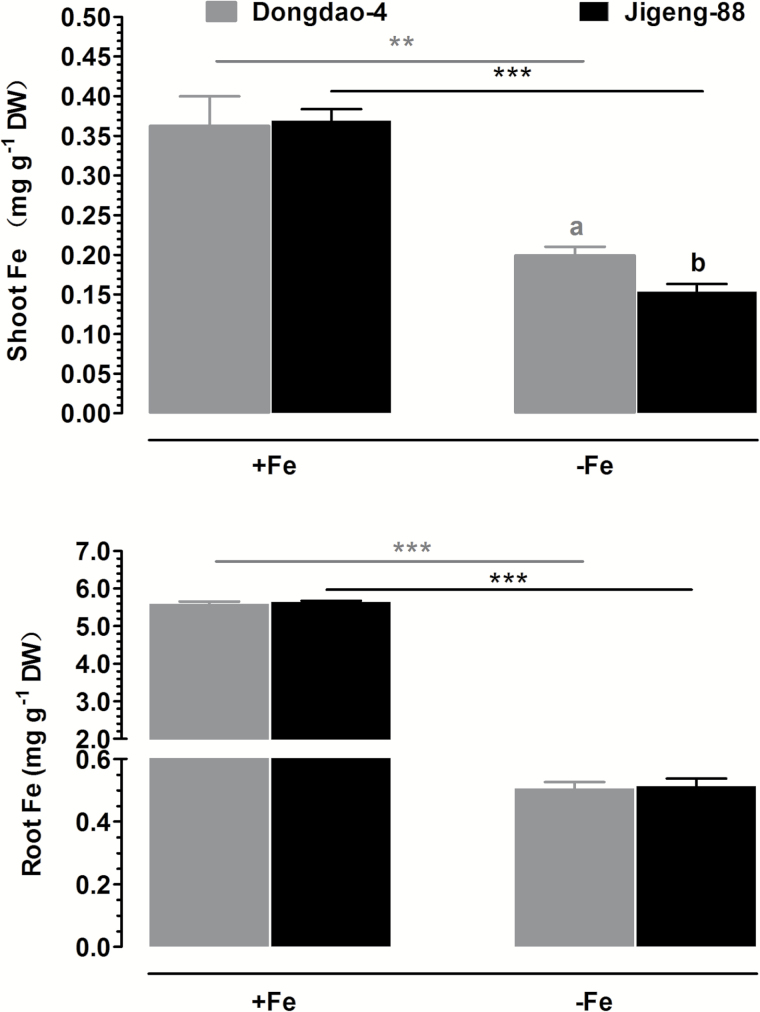
Fe concentration in (A) shoots and (B) roots of Dongdao-4 and Jigeng-88 seedlings grown in Fe-sufficient or Fe-deficient conditions. Treatments and statistical analysis were as described in Fig. 9.

We further compared Fe concentrations in shoots and roots of the two genotypes under Fe-sufficient and Fe-deficient conditions. Under Fe-sufficient conditions, there was no significant difference between the two genotypes in shoot Fe concentration (Fig. 10A). After exposure to Fe-deficient medium there were significant reductions in shoot Fe concentrations in both genotypes; the reduction in shoot Fe concentration in Jigeng-88 was greater than that in Dongdao-4, leading to a significantly higher shoot Fe concentration in Dongdao-4 *versus* Jigeng-88 plants (Fig. 10A). Root Fe concentrations in the two genotypes were comparable under Fe-sufficient conditions; exposure to Fe-deficient medium led to similar reductions in root Fe concentration in both genotypes (Fig. 10B).

### Effect of Fe deficiency on rhizosphere acidification

**Fig. 11.**
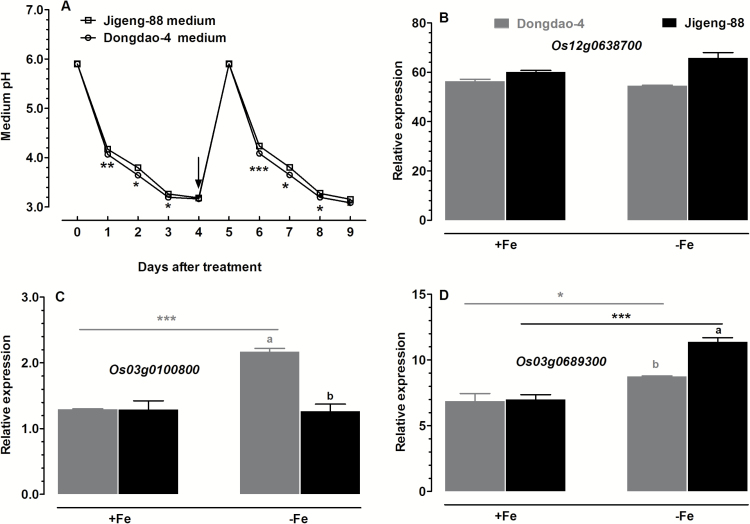
Effects of different levels of Fe supplementation on root acidification. (A) Time course of changes in the pH of the growth solution following exposure of plants previously grown in Fe-sufficient (+Fe) medium to Fe-deficient (–Fe) medium for 9 days. The arrow indicates the time when the growth medium was renewed; at the time of medium renewal, the pH was adjusted to 5.9. (B–D) Expression patterns of the plasma membrane H^+^-ATPase genes (B) *Os12g0638700*, (C) *Os03g0100800*, and (D) *Os03g0689300* in roots of Dongdao-4 and Jigeng-88 plants exposed to +Fe and –Fe medium for 9 days. Treatments and statistical analysis were as described in Fig. 9.

Upon exposure to Fe-deficient medium, there were significant reductions in medium pH for both rice genotypes (Fig. 11). The Fe-deficiency-induced reduction in medium pH of Dongdao-4 was greater than that of Jigeng-88, resulting in a significantly higher medium pH for Jigeng-88 (Fig. 11A). These results indicate that Fe-deficiency-induced acidification is greater in Dongdao-4 than in Jigeng-88 plants (Fig. 11A).

We further monitored changes in the expression of the H^+^-ATPase-encoding genes *Os12g0638700*, *Os03g0100800*, and *Os03g0689300* in the two genotypes. Levels of expression of *Os12g0638700* were comparable in the two genotypes under both Fe-sufficient and Fe-deficient conditions (Fig. 11B). Under Fe-sufficient conditions, expression levels of *Os03g0100800* and *Os03g0689300* were comparable in Dongdao-4 and Jigeng-88 plants (Fig. 11C, D). The expression of *Os03g0100800* was markedly up-regulated by exposure to Fe deficiency in Dongdao-4 plants, whereas the expression level in Jigeng-88 plants exposed to Fe-deficient medium remained relatively unchanged, leading to significantly higher expression levels in Dongdao-4 plants (Fig. 11C). Expression of *Os03g0689300* was markedly up-regulated by Fe deficiency in both genotypes; the up-regulation was more pronounced in Jigeng-88 plants, such that significantly higher expression in Jigeng-88 *versus* Dongdao-4 plants was observed in Fe-deficient conditions (Fig. 11D).

### Effect of Fe deficiency on secretion of phytosiderophores

**Fig. 12.**
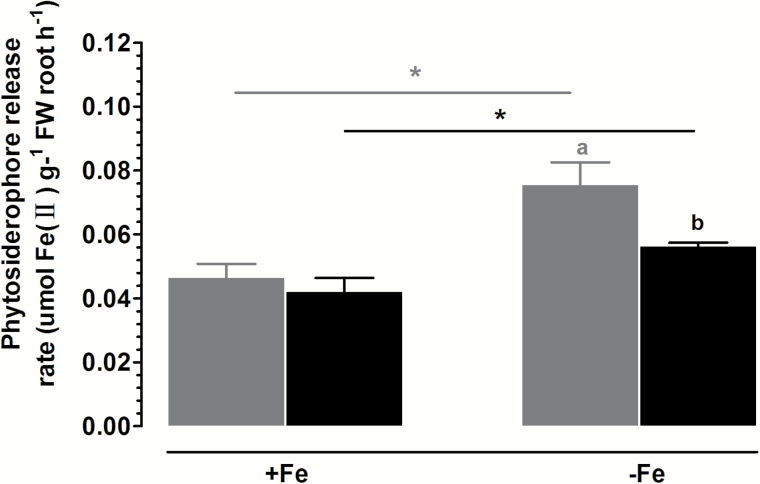
Effects of Fe deficiency on the rate of phytosiderophore secretion from roots of Dongdao-4 and Jigeng-88 plants. Data are means±SE (*n*=4). Treatments and statistical analysis were as described in Fig. 9.

As shown in Fig. 12, no difference between the two genotypes in the rate of phytosiderophore secretion from the roots was detected under Fe-sufficient conditions. Exposure to Fe-deficient medium led to a significantly greater increase in phytosiderophore release from Dongdao-4 than from Jigeng-88 roots (Fig. 12).

### Effects of Fe deficiency on expression levels of Fe deficiency-responsive genes

**Fig. 13.**
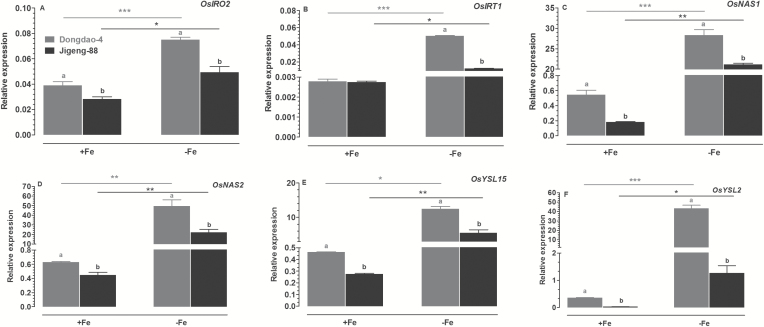
Effects of Fe deficiency on the expression of six Fe-deficiency-responsive genes in roots of Dongdao-4 and Jigeng-88 plants: (A) *OsIRO2*, (B) *OsIRT1*, (C) *OsNAS1*, (D) *OsNAS2*, (E) *OsYSL15*, and (F) *OsYSL2*. Total RNA was extracted from rice seedlings grown under Fe-sufficient or Fe-deficient medium for 1week. Transcript levels were measured by real-time RT-PCR. Actin was used as an internal control. Error bars are calculated based on three biological replicates. Treatments and statistical analysis were as described in Fig. 9.

To investigate the mechanisms underlying the different tolerance of the two genotypes to Fe deficiency, we monitored changes in the expression of *OsIRO2, OsIRT1, OsNAS1, OsNAS2, OsYSL15*, and *OsYSL2* in roots. Under Fe-sufficient conditions, the levels of expression of *OsIRT1* in roots of Dongdao-4 and Jigeng-88 plants were comparable (Fig. 13A). When treated with Fe-deficient medium, the expression level of *OsIRT1* was up-regulated in both genotypes; the increase was greater in Dongdao-4 than in Jigeng-88 (Fig. 13B). The levels of expression of *OsIRO2, OsNAS1, OsNAS2, OsYSL15*, and *OsYSL2* in both genotypes were also up-regulated by exposure to Fe-deficient medium (Fig. 13A, C–F). Similar to *OsIRT1*, the magnitude of Fe deficiency-induced increases in expression of *OsIRO2, OsNAS1, OsNAS2, OsYSL15* and *OsYSL2* in Dongdao-4 plants were higher than those in Jigeng-88 plants (Fig. 13A, C–F).

## Discussion

Dongdao-4 is an elite rice genotype that is capable of growing in saline-alkaline soils in the northeast of China. However, the physiological and molecular mechanisms underlying the tolerance of Dongdao-4 plants to saline-alkaline stress remain largely unknown. In the present study, we evaluated the effect of saline-alkaline stress on Dongdao-4 plants and on a relatively saline-alkaline-sensitive genotype, Jigeng-88. Our results demonstrated that Dongdao-4 plants were more tolerant to saline-alkaline stress than Jigeng-88 plants, as evidenced by less marked reductions in plant height and shoot biomass (Fig. 1). The greater biomass of Dongdao-4 plants may be accounted for by their higher chlorophyll concentration and photosynthetic rates than those in Jigeng-88 plants under saline-alkaline conditions. We further showed that Dongdao-4 plants had a higher shoot Na^+^/K^+^ ratio than Jigeng-88 in both control and saline-alkaline media. One important finding in the present study is that Dongdao-4 plants can maintain a higher shoot Fe concentration and a larger root system than Jigeng-88 plants under saline-alkaline conditions; these findings suggest that Dongdao-4 is a Fe-efficient genotype, as low Fe availability in saline-alkaline soil is a limiting factor for rice growth. Finally, we provide experimental evidence to support the hypothesis that Dongdao-4 plants were more tolerant to Fe deficiency than Jigeng-88 plants. These findings highlight that maintenance of a higher shoot Fe concentration and a larger root system are important strategies for rice genotypes tolerant to saline-alkaline stress.

Plants suffering from saline-alkaline stress have to cope with toxic concentrations of Na^+^ in the growth medium. Plants with high tolerance to saline stress often maintain a high K^+^ and low Na^+^ concentration in their shoots. Thus, Na^+^/K^+^ ratio is often regarded as an indicator of plant tolerance to saline stress (Zhu, 2003). However, there are reports demonstrating independence of salt tolerance and Na^+^ accumulation among wheat genotypes (Genc *et al.*, 2007). For example, Babgohari *et al.* (2013) found that salt- tolerant wheat exhibited a higher Na^+^/K^+^ ratio than the salt-sensitive genotype. In addition, Tang *et al.* (2014) showed that transgenic poplar plants overexpressing *PtCBL10s* had greater tolerance to saline stress and accumulated higher Na^+^ in stems compared with wild-type plants when grown in salt-supplemented solution (Tang *et al.*, 2014). Recently, Wang *et al.* (2016) reported that heterologous expression of *PtCYP714A3*, a cytochrome P450 monooxygenase gene from *Populus trichocarpa*, enhanced tolerance to salt stress in transgenic rice. Compared with wild-type plants, the transgenic rice maintained a higher Na^+^/K^+^ ratio in shoots under control and salt-stress conditions (Wang *et al.*, 2016). In the present study, we found that Dongdao-4 plants exhibited a higher Na^+^/K^+^ ratio in shoots and roots than Jigeng-88 plants when grown under control conditions (Fig. 3C, F), suggesting that Dongdao-4 plants tend to accumulate more Na^+^ under normal conditions. When exposed to saline-alkaline treatment, Dongdao-4 plants exhibited better growth performance than Jigeng-88 plants. However, a higher Na^+^/K^+^ ratio was observed in shoots of Dongdao-4 *versus* Jigeng-88 in saline-alkaline medium (Fig. 3C), suggesting that the tolerance of Dongdao-4 plants to saline-alkaline stress is not achieved by minimizing Na accumulation. Excessive accumulation of Na^+^ is toxic to many enzymes in the cytosol (Zhu, 2001). Plants can sequester Na^+^ in the vacuole via Na^+^/H^+^ antiporters in the tonoplast, thus minimizing its toxic effect under salt stress (Bassil and Blumwald, 2014; Orlowski and Grinstein 1997). In addition, Na^+^ compartmentation can facilitate water uptake from saline medium by osmotic adjustment because Na^+^ can be used as an osmolyte in the vacuole to maintain a water potential gradient for water influx into root cells (Zhu, 2001). Therefore, compartmentalization of Na into vacuoles is an essential strategy for salt tolerance in plants. There have been several reports showing that overexpression of *NHX* led to improved salt tolerance in a range of diverse species (Apse *et al.*, 1999; Fukuda *et al.*, 2004; Liu *et al.*, 2008; Ohta *et al.*, 2002; Rodríguez-Rosales *et al.*, 2008). Dongdao-4 also exhibited lower K^+^ concentrations in shoots than Jigeng-88 plants under both control and saline-alkaline conditions (Fig. 3), suggesting that Dongdao-4 plants may have a low requirement for K^+^ to maintain their growth.

In addition to the presence of high levels of toxic Na^+^ in saline-alkaline medium, high pH, due to the hydrolysis of NaHCO_3_ and Na_2_CO_3_, can have a substantial impact on the availability of many mineral nutrients in the soil solution, thus exposing plants to nutrient stress (Shi and Wang, 2005). In saline-alkaline soils, Fe occurs mainly in the forms of insoluble hydroxides and oxides, limiting its bioavailability for plants (Romera and Alcántara, 2004). In the present study, mixtures of NaCl and Na_2_CO_3_ were added into the nutrient solution to mimic the concentrations that occur in saline-alkaline soil with a high Na^+^ concentration and high soil pH. It has been reported that buffering pH to 8.3 leads to substantial precipitation of Fe along with other nutrients in a mural habitat (Donnini *et al.*, 2012). In the current study, we also observed Fe precipitation from the saline-alkaline solution, resulting in a lower available Fe concentration (See Supplementary Table S1 at JXB online). Therefore, the rice plants growing in the saline-alkaline medium had to cope with low availability of Fe in addition to Na^+^ toxicity. The less inhibitory effect of saline-alkaline stress on the growth of Dongdao-4 plants compared with Jigeng-88 plants may imply that Dongdao-4 plants have a more efficient system for Fe acquisition. To test this hypothesis, we measured Fe concentrations in the shoots and roots of the two genotypes under control and saline-alkaline conditions. There was a significant reduction in the Fe concentration in shoots of Jigeng-88 seedlings upon exposure to saline-alkaline medium, whereas the Fe concentration in shoots of Dongdao-4 seedlings remained relatively unchanged, leading to a significantly higher Fe concentration in Dongdao-4. These results again imply that Dongdao-4 plants may be equipped with an efficient Fe-acquisition system, which provides them with tolerance to the Fe deficiency evoked by saline-alkaline stress.

Characteristics of the root system are important for efficient Fe acquisition in plants growing under Fe-deficient conditions. The observation that Dongdao-4 seedlings had a higher Fe concentration in their leaves than Jigeng-88 plants under saline-alkaline conditions prompted us to evaluate whether their root systems differed in response to saline-alkaline treatment. The root traits of the two genotypes were identical under control conditions, but Dongdao-4 seedlings exhibited a significantly higher root surface area and larger adventitious root number under saline-alkaline conditions. These results suggest that the more extensive root system of Dongdao-4 seedlings may facilitate Fe acquisition, thus equipping them with tolerance to the Fe deficiency associated with saline-alkaline stress.

Despite its abundance in soils, Fe is predominantly present in insoluble oxidized Fe^3+^-compound form in alkaline soil, and thus the available Fe to plants in such soils is low. To cope with low Fe availability, higher plants have evolved two distinct strategies (I and II) to acquire Fe from the rhizosphere (Guerinot and Yi, 1994). In the present study, we found that Dongdao-4 plants showed stronger rhizosphere acidification than Jigeng-88 plants, and that the levels of expression of three H^+^-ATPase-encoding genes were higher in Dongdao-4 than in Jigeng-88 under both saline-alkaline and Fe-deficient conditions (Figs 6 and 11). The greater capacity of Dongdao-4 to acidify the rhizosphere due to enhanced expression of genes encoding H^+^-ATPases may contribute to the higher Fe acquisition efficiency of this genotype under saline-alkaline and Fe-deficient conditions. In contrast to proton exudation, no significant differences between the two genotypes in ferric chelate reductase activities were detected under control or saline-alkaline conditions (Supplementary Fig. S1); this finding suggested that, under our experimental conditions, ferric chelate reductase activity is not involved in the greater tolerance of Dongdao-4 to saline-alkaline stress.

As rice is a Strategy II plant, the synthesis and exudation of phytosiderophores to chelate Fe^3+^ and the uptake of Fe^3+^–MAs complexes via specific transporters are key means of Fe acquisition by rice plants in Fe-deficient conditions (Araki *et al.*, 2015; Jolley and Brown, 1989). In this study, we found that the rate of exudation of phytosiderophores in Dongdao-4 plants was greater than that in Jigeng-88 plants under saline-alkaline conditions (Fig. 7). The higher rate of exudation of phytosiderophores from Dongdao-4 plants would allow more efficient chelation of Fe^3+^, thus providing them with greater tolerance to low availability of Fe relative to Jigeng-88 plants. In this context, an enhanced secretion of phytosiderophores by rice plants in response to Fe deficiency in calcareous soils has been reported (Higuchi *et al.*, 1999).

To further explore the molecular mechanisms underlying the greater accumulation of Fe in Dongdao-4 plants compared with Jigeng-88 plants, we monitored changes in the expression patterns of genes involved in Fe acquisition. OsIRT1 is a Fe^2+^ transporter for direct uptake of Fe from soil, which is expressed in the epidermal cells of Fe-deficient roots (Ishimaru *et al.*, 2006). In calcareous soils with high pH, Fe-deficient barley plants exude phytosiderophores from their roots to chelate Fe^3+^ from the rhizosphere (Awad *et al.*, 1988). NAS, which is encoded by *OsNAS1* and *OsNAS2*, is a key enzyme in the biosynthesis of the MA family of phytosiderophores (Higuchi *et al.*, 1999); *OsNAS1* and *OsNAS2* are differentially regulated by Fe deficiency. OsYSL15 is the primary transporter in rice responsible for the uptake of Fe^3+^–MA complexes from the rhizosphere (Inoue *et al.*, 2009). Kobayashi *et al.* (2015) found that homologs of the genes involved in Fe acquisition (including *NAS*, *NAAT*, and *YSL*) were up-regulated in *P. tenuiflora* and down-regulated in *P. annua* when challenged by NaHCO_3_. In addition, Gómez-Galera *et al.* (2012) reported that constitutive expression of a barley Fe phytosiderophore transporter (*HvYS1*) confers tolerance to alkaline soil, due to Fe mobilization. Murata *et al.* (2015) reported that expression of *HvYS1*, a Fe^3+^-phytosiderophore transporter gene, conferred tolerance to Fe deficiency in transgenic petunia in alkaline environments (Murata *et al.*, 2015). *OsIRO2* is a basic helix-loop-helix transcription factor induced by Fe deficiency (Ogo *et al.*, 2006), which is responsible for regulating the genes involved in Fe homeostasis in rice, including *OsNAS1*, *OsNAS2*, and *OsYSL15* (Kobayashi and Nishizawa, 2012). Ogo *et al.* (2011) reported that OsIRO2 plays a role in the improvement of rice growth and yield by enhancing Fe utilization in calcareous soils. In the current study, the expression levels of the genes *OsIRO2*, *OsIRT1*, *OsNAS1*, *OsNAS2*, *OsYSL15*, and *OsYSL2* in the two rice genotypes were markedly enhanced by saline-alkaline treatment, and the magnitude of the up-regulation was greater in Dongdao-4 than in Jigeng-88. These results suggest that the higher expression levels of these genes may allow Dongdao-4 plants to acquire Fe more efficiently, thus contributing to their greater accumulation of Fe when exposed to saline-alkaline stress.

The observation that Dongdao-4 plants can better maintain Fe homeostasis under saline-alkaline conditions relative to Jigeng-88 implies that the Dongdao-4 genotype is highly efficient for Fe acquisition. We further tested this hypothesis by comparing the effects of Fe deficiency on Dongdao-4 and Jigeng-88 plants. Consistent with our hypothesis, we found that Dongdao-4 plants exhibited greater tolerance to low Fe availability (exposure to Fe-deficient growth medium) than Jigeng-88 plants. Taken together, the more extensive root system, more pronounced rhizosphere acidification, higher phytosiderophore release rate and higher level of expression of Fe-deficiency-related genes in Dongdao-4 seedlings may facilitate their acquisition of Fe, thus providing greater tolerance to Fe-deficiency stress.

In summary, this study demonstrated that Dongdao-4, an elite rice genotype that was bred in saline-alkaline soil in northern China, was more tolerant to saline-alkaline stress and Fe deficiency than the rice genotype Jigeng-88. When grown in both Fe-deficient and saline-alkaline conditions, Dongdao-4 plants can accumulate more Fe than Jigeng-88 plants due to their larger root systems and higher expression levels of Fe-deficiency-related genes, which allow Dongdao-4 plants to acquire Fe more efficiently under the conditions of low availability of Fe in soil. These findings will be valuable for dissecting the physiological and molecular mechanisms associated with the responses of rice to saline-alkaline stress and Fe deficiency.

## Supplementary data

Supplementary data are available at *JXB* online.

Table S1. Effects of saline-alkaline stress on availabilities of mineral nutrients in the growth medium.

Fig. S1. Effects of saline-alkaline stress on root ferric chelate reductase activity.

Fig. S2. Effects of Fe-deficient conditions on root ferric chelate reductase activity.

Supplementary Data
