# Supplementary material for: Efficient acquisition of iron confers greater tolerance to saline-alkaline stress in rice (Oryza sativa L.)
Source: J Exp Bot. 2016 Nov 3;67(22):6431–44. doi: 10.1093/jxb/erw407 (PMC5181582; doi:10.1093/jxb/erw407)
Supplement: Supplementary Data [file supp_67_22_6431__index.html]

Efficient acquisition of iron confers greater tolerance to saline-alkaline stress in rice (Oryza sativa L.) — Efficient acquisition of iron confers greater tolerance to saline-alkaline stress in rice (Oryza sativa L.) — Supplementary Data 

# Efficient acquisition of iron confers greater tolerance to saline-alkaline stress in rice (*Oryza sativa* L.)

## Supplementary Data

Data files

- supplementary\_figures\_S1\_S2.pdf - Supplementary Data
- supplementary\_table\_S1.pdf - Supplementary Data
