# Supplementary material for: Efficient acquisition of iron confers greater tolerance to saline-alkaline stress in rice (Oryza sativa L.)
Source: J Exp Bot. 2016 Nov 3;67(22):6431–44. doi: 10.1093/jxb/erw407 (PMC5181582; doi:10.1093/jxb/erw407)
Supplement: Supplementary Data [file supp_erw407_supplementary_figures_S1_S2.pdf]

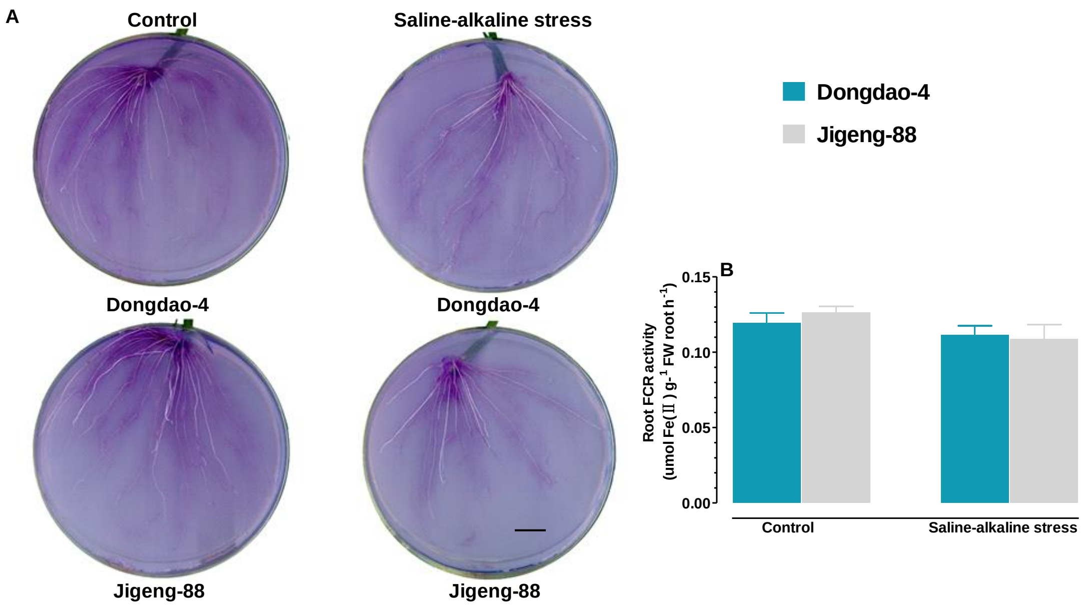

Figure S1. Effects of saline-alkaline stress on root ferric chelate reductase (FCR) activity. (A) Visualization of FCR activity in roots of Dongdao-4 and Jigeng-88 plants which grown under control and saline-alkaline stress conditions. After 5 day cultivation in the nutrient solution with and without saline-alkaline stress, the rice seedlings were incubated in agar medium containing the ferrous chelate ferrozine. The purple colour indicates that ferric was reduced to ferrous by ferric reductase and chelate by ferrozine. The experiment was performed twice with similar results with n=5. Bar, 2 cm. (B) Ferric chelate reductase activity.

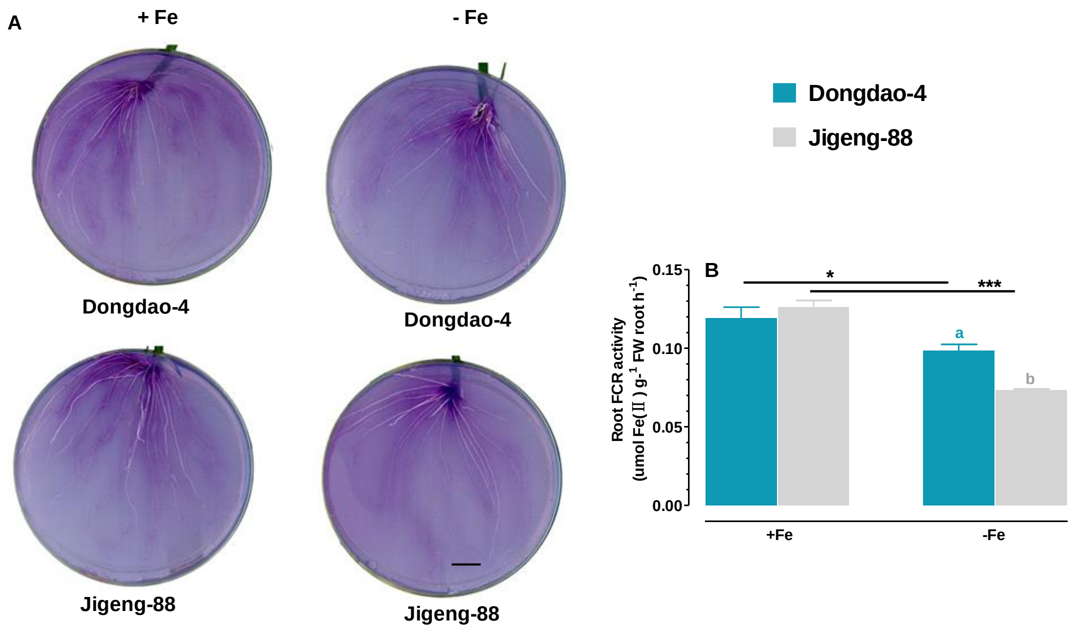

Figure S2. Effects of Fe-deficient on root ferric chelate reductase (FCR) activity. (A) Visualization of FCR activity in roots of Dongdao-4 and Jigeng-88 plants which grown in different Fe levels. After 7 day cultivation in Fe-sufficient and Fe-deficient condition, the rice seedlings were incubated in agar medium containing the ferrous chelate ferrozine. The purple colour indicates that ferric was reduced to ferrous by FCR and chelate by ferrozine. The experiment was performed twice with similar results with n=5. Bar, 2 cm. (B) Ferric chelate reductase activity. Means with different letters are significantly different (P<0.05) within the same treatments. Asterisks indicate significant differences between control and saline-alkali stress of the same genotype determined by Student's t-test (*0.01<P<0.05, *** P<0.001).
