# Supplementary material for: Efficient acquisition of iron confers greater tolerance to saline-alkaline stress in rice (Oryza sativa L.)
Source: J Exp Bot. 2016 Nov 3;67(22):6431–44. doi: 10.1093/jxb/erw407 (PMC5181582; doi:10.1093/jxb/erw407)
Supplement: Supplementary Data [file supp_erw407_supplementary_table_S1.pdf]

Table S1. Effects of saline-alkaline stress on the concentrations of various ions in nutrient solution.

	Fe (mM)	Zn (μM)	Mg(mM)	Mn (μM)	Cu (μM)	Ca (mM)
Control	0.13	0.15	1.64	9.45	0.15	0.73
Saline-alkaline	0.06	0.05	1.57	7.70	0.11	0.64
